# Prevalence and associated factors of eight antenatal care contacts among mothers who give birth in Shebel Berenta district, East Gojjam zone, northeast Ethiopia, 2024

**DOI:** 10.3389/frph.2025.1531380

**Published:** 2025-08-11

**Authors:** Nigusie Abebaw, Yezbalem Negesse, Asnake Begashw, Dagnachew Amare, Genet Tsegaw, Dessalew Meshesha, Kefale Geto

**Affiliations:** ^1^ Department of Midwifery, College of Medical Health Science, Wollo University, Dessie, Ethiopia; ^2^ Department of Midwifery, Mekidela Amba Primary Hospital, Mekidela, Ethiopia; ^3^ Department of Midwifery, Mida Weremo Primary Hospital, Meragna, Ethiopia; ^4^ Department of Midwifery, Dessie Health Science College, Dessie, Ethiopia; ^5^ Department of Midwifery, Sayint Primary Hospital, Sayint, Ethiopia; ^6^ Department of Midwifery, Shebel Berenta Health Office, Shebel Berenta, Ethiopia

**Keywords:** eight contacts, pregnant women, Ethiopia, antenatal care, associated factors

## Abstract

**Introduction:**

Antenatal care refers to the medical attention provided by skilled healthcare professionals to pregnant women to ensure optimal health outcomes for both the mother and fetus throughout pregnancy. In Ethiopia, there is limited evidence regarding the completion of eight antenatal care contacts and the factors associated with it. Therefore, the aim of this study was to assess the prevalence and associated factors of completing eight antenatal care contacts among mothers in Shebel Berenta Woreda.

**Method:**

A community-based cross-sectional study was employed. A stratified sampling technique was employed to select the sample. Data were exported from Kobo Toolbox software to SPSS version 27 for analysis. Bivariable and multivariable logistic regression analyses were used to assess associations between the outcome and independent variables, with statistical significance determined at a *p*-value of <0.05.

**Result:**

This study showed that the prevalence of eight or more antenatal care contacts was 9.6% [95% confidence interval (CI): 7.4–12.3]. In this study, good knowledge of ANC [adjusted odds ratio (AOR) = 2.402; 95% CI: 1.115–5.175], media exposure (AOR = 2.47; 95% CI: 1.15–5.306), time of early initiation for first ANC contact (AOR = 5.46; 95% CI: 2.837–10.51), women's governmental occupation (AOR = 3.745; 95% CI: 1.364–10.28), two to four pregnancies (AOR = 3.524; 95% CI: 1.696–7.32), and family size less than five (AOR = 3.005; 95% CI: 1.461–6.179) were significantly associated with the outcome variable.

**Conclusion:**

The study indicates that the prevalence of eight or more antenatal care contacts was low. Time of initiation for first ANC contact, women's occupational status, knowledge of antenatal care, family size, number of pregnancies, and media exposure were significantly associated with the outcome variable.

## Introduction

Antenatal care (ANC) is a broad term to describe the medical attention provided by skilled healthcare professionals for pregnant women to ensure optimal health conditions for both the mother and fetus throughout pregnancy (1). Antenatal care is a vital service for reducing pregnancy complications and improving child health outcomes (2, 3).

**Table 1:** Sample size determination for assessing the prevalence and associated factors of eight antenatal care contacts among mothers in Shebel Berenta District, East Gojjam Zone, northeast Ethiopia, 2024.

Variables	Outcome	AOR		Sample size	Reference
		Exposed (%)	Unexposed (%)		
Age of the mother	9.5	25.1	1.83	208	(28)
Wealth index	4.7	14	1.67	348	(28)
Educational status of the women	14.4	1.7	1.588	172	(26)
Partner educational status	12.7	2.2	1.471	232	(26)
Maximum sample size	348				

Finally, the largest sample size was found to be 348. Considering a design effect of 1.5 and adding a non-response rate of 10%, the final sample size was 574.

Antenatal care is essential to maintain the health of women and their unborn children. Women can learn healthy behaviors during pregnancy and better recognize danger signs during pregnancy and childbirth. Through antenatal care, pregnant women can also access micronutrient supplementation, such as iron-folic acid, treatment for hypertension to prevent eclampsia, immunization against tetanus, human immunodeficiency virus (HIV) testing, and in areas where malaria is endemic, medications and insecticide-treated mosquito nets (2, 4–6). ANC facilitates and increases opportunities for the uptake of preventive measures, the timely detection of danger signs, a reduction in complications, and addressing healthcare inequalities (7).

The World Health Organization (WHO) recommends at least eight ANC contacts during pregnancy, replacing the previous focused antenatal care model. These include one contact in the first trimester (up to 12 weeks), two in the second trimester (at 20 and 26 weeks), and five contacts in the third trimester (at 30, 34, 36, 38, and 40 weeks) to improve perinatal outcomes and enhance women's experience of care (8).

Globally, maternal mortality is alarmingly high. In 2020, 287,000 women died during and after pregnancy and childbirth. These deaths predominantly occurred in low- and lower-middle-income countries, accounting for approximately 95% of all maternal fatalities. Tragically, the majority of these deaths could have been prevented. Sub-Saharan Africa (SSA) alone accounted for 70% of global maternal deaths. Ethiopia also contributed significantly in 2020, with 3.6% of global maternal deaths (9). ANC remains a prominent measure for improving maternal health outcomes (10).

Ethiopia acknowledges the importance of antenatal care in improving maternal and neonatal health outcomes. However, the benefits of ANC are only realized when expectant mothers use these services as recommended. In many underdeveloped countries, the prevalence of ANC service utilization remains low. Regular prenatal visits are essential for the early identification and management of maternal complications and risk factors (11).

A higher frequency of ANC contacts with healthcare providers can reduce maternal mortality by up to 20% during pregnancy, labor, delivery, and the postnatal period. It also plays a vital role in reducing the incidence of stillbirth, neonatal and infant mortality, stunting, and underweight (4, 7, 12–14). High-quality ANC follow-up can lower stillbirth rates by almost half—both directly through preventative care and indirectly by encouraging deliveries in health facilities where complications can be effectively managed (4). Receiving a minimum of eight ANC contacts before delivery has been shown to reduce the risk of preterm birth and low birth weight by 72% and 64%, respectively (15). Similarly, eight antenatal care visits are associated with a 45.2% reduction in the likelihood of under-five mortality (16, 17).

The increased risk of fetal death between 32 and 36 weeks of gestation may be linked to a reduced number of antenatal visits. Asymptomatic conditions such as pre-eclampsia, fetal growth restriction, and unexplained intrauterine death may present unexpectedly in the third trimester. More frequent routine visits during this period can help detect these complications earlier, allowing for timely intervention (18). A study based on 54 Demographic and Health Surveys (DHS) from low- and middle-income countries found that only 11.3% of women received eight or more ANC contacts (19). In 2021, the prevalence of compliance with the eight-visit ANC model in SSA was 7.7% (20).

Although Ethiopia adopted the 2016 WHO model recommending eight antenatal care contacts in 2020 to reduce maternal and perinatal mortality and morbidity (1, 21), implementation remains limited. The guidelines focus on key ANC principles, including pregnant-woman-centered care, maternal and fetal assessments during initial and follow-up contacts, prevention and treatment of common pregnancy-related issues, counseling and health promotion, as well as strengthening the health system to improve ANC coverage (1).

Although several studies have examined the prevalence and associated factors of eight or more antenatal care contacts, there remains a gap in understanding the specific role of women's knowledge and attitudes toward these contacts. In addition, most of the research relies on secondary data, and no studies have been conducted in the current study area.

Therefore, the aim of the present study was to assess the prevalence and factors associated with receiving eight or more ANC contacts among mothers in Shebel Berenta Woreda, Ethiopia. ANC is crucial for reducing maternal mortality and improving maternal and child health outcomes.

## Method and materials

### Study area and period

Shebel Berenta Woreda is located in East Gojjam Zone, situated in the north central highlands of Ethiopia in Amhara National Regional State. It extends from 10° 15′ N to 10° 30′ N latitude and from 38° 15′ E to 38° 27′ longitude. It is found 293 km northeast of Addis Ababa, the capital city of Ethiopia. Shebel Berenta Woreda has 26 kebeles (4 urban and 22 rural kebeles). Shebel Berenta Woreda has 1 primary hospital, 6 health centers, and 24 health posts. According to the Central Statistics Agency of Ethiopia’s population projection of Woreda in 2023, the total population is 130,412, of which 63,634 are males and 66,778 are females (21). Over the past 6 months 1,683 mothers have given birth, according to the woreda report.

The study was conducted between 15 April and 31 May 2024.

### Study design

A community-based cross-sectional study was employed.

### Source population

All mothers who gave birth in the last 6 months in Shebel Berenta District were the source population.

### Study population

Mothers who gave birth in the last 6 months in selected kebeles of Shebel Berenta District during the data collection period comprised the study population.

#### Inclusion criteria

Mothers residing in Shebel Berenta District who gave birth within the last 6 months before data collection were included.

#### Exclusion criteria

Mothers who recently migrated to Shebel Berenta District after childbirth and those unable to provide information during the data collection period were excluded.

### Sample size determination

The sample size for each objective was determined using the StatCalc function in OpenEPI Info software version 7.2.6, and the largest calculated sample size was selected for this study (Table 1).

To determine the prevalence of eight antenatal care contacts, the single population proportion formula was used, based on the following assumptions:

$$\mathrm{Using}\;\mathrm{the}\;\mathrm{formula}:\;N=\frac{{\left(Z_{\frac{\alpha }{2}}\right)}^{2}pq}{d^{2}}$$

where *N* is the minimum sample size required for the study, *Z* is the standard normal distribution (*Z* = 1.96) with a 95% confidence interval (CI), *P* is the prevalence of eight ANC contacts (30.7% in Addis Ababa) (12), and *d* is a tolerable margin of error (*d* = 0.05). Therefore, the sample size for the first objective is 327.

### Sampling technique

To ensure a comprehensive understanding of the prevalence and associated factors of eight or more ANC contacts in Shebel Berenta District, a stratified sampling method was employed. Initially, the population was divided into two strata based on urban and rural settings, given the district's mix of both. A total of 4 urban and 22 rural kebeles were identified. Stratified sampling was used to ensure adequate representation of both urban and rural populations. Of the kebeles, 30% (eight rural and two urban) were randomly selected from each stratum. With each selected kebele, a list of mothers who had given birth in the past 6 months was obtained from health extension workers. Simple random sampling within the selected kebele was then implemented to select households or individuals using the lottery method. If the selected participant was not present during data collection, at least two revisits were made to interview her on the other day. If she was still unavailable, the next eligible woman was selected and interviewed.

### Data collection procedure

Data were collected using a pre-tested semi-structured questionnaire through the Kobo Collect mobile application. The process was conducted by four diploma-level midwives under the supervision of two BSc midwives. The principal investigator oversaw the overall data collection process. Both the data collectors and supervisors were trained by the principal investigator. Only mothers who were willing to participate and had signed the informed consent form were interviewed.

### Data quality control

The data collection tool was primarily prepared in the English language. It was then translated into the local language (Amharic). Finally, it was re-translated back into English to check its accuracy and consistency. The questionnaire was pre-tested on a 5% sample size from Mankorkuay Kebele in Enemay Woreda. Training was provided to data collectors and supervisors both 1 day before and 1 day after the pretest. The training covered the objectives of the study, the data collection tool, procedures for data collection, and methods for ensuring the completeness of the data. To maintain data quality, proper coding and categorization were implemented. Each day, the principal investigator and supervisors reviewed the collected data for completeness, accuracy, clarity, and consistency. The reliability of the Likert scale variables was assessed using Cronbach's alpha (0.807).

#### Dependent variables

The dependent variables were the eight and above ANC contacts.

#### Independent variables

##### Sociodemographic characteristics

Sociodemographic characteristics included maternal age, maternal and husband’s educational status, maternal occupation, marital status, wealth index, residence, religion, type of family, family size, age at marriage, and health insurance coverage.

##### Obstetrics characteristics

Obstetric characteristics included number of pregnancies, parity, place of delivery, contraceptive use, birth interval, birth order, whether the pregnancy was planned, experience of pregnancy-related complications, timing of ANC initiation, and awareness of pregnancy complications.

##### Health service-related factors

Health service-related factors included the type of institution being attended (private or governmental).

##### Information and decision-making-related characteristics

Information and decision-making-related characteristics included media exposure, the primary decision-maker regarding maternal healthcare, and knowledge and attitudes toward ANC contacts.

### Operational definitions

#### Media exposure

Media exposure was obtained by aggregating women's exposure to television, radio, and newspapers. Women were considered exposed if they accessed any of these media at least once a week (22).

### Measurement of household wealth index

The household wealth index, used as a proxy indicator for socioeconomic status, was calculated using principal component analysis (23).

#### Knowledge of ANC

The minimum and maximum scores for knowledge of ANC were 0 and 18, respectively. Based on the total score from the knowledge assessment variables, participants scoring above the mean were considered knowledgeable, while those scoring below the mean were classified as having poor knowledge (24).

#### Attitude toward ANC

The total attitude score toward ANC was in the range of 8–40. Women scoring above the mean were considered to have a good attitude (24).

### Data processing and analysis

Data were collected through the Kobo Tools app and then exported to Statistical Package for Social Sciences (SPSS) software version 27 for processing and analysis. Descriptive statistics for different variables were presented as frequency, percentage tables, and pie charts. Logistic regression was used to identify variables significantly associated with the outcome. Both bivariable and multivariable logistic regressions were performed to examine associations between independent variables and the outcome. Variables with a *p*-value <0.25 in the bivariate analysis were included in the multivariable model. Multi-collinearity among independent variables was assessed using the variance inflation factor (1.17) and tolerance tests. In the multivariable analysis, adjusted odds ratios (AORs) with 95% CIs were used to determine the strength of the association. Variables with *p*-values <0.05 were considered statistically significant. The model's fitness was confirmed by the Hosmer–Lemeshow test (0.427), indicating a good fit for the logistic regression model.

A principal component analysis was employed to determine the household wealth index. Initially, wealth index variables were selected, then descriptive statistics and standard deviations were calculated. Variables that met the assumptions of principal component analysis were retained, and wealth index quintiles were created by ranking the cases.

### Ethical considerations

Ethical clearance was obtained from Wollo University, College of Medicine and Health Sciences, on behalf of the Research Ethics Review Committee (reference number CMHS/519/2024). A formal letter requesting permission and support was sent from the Wollo University Department of Midwifery to the Shebel Berenta Woreda Health Office, and from there to each selected kebele leader. All study participants were informed about the purpose, risks, benefits, and confidentiality of the study, as well as their right to refuse participation. Written and signed voluntary consent was obtained from all participants before the interview. Respondents were also assured that the information they provided would be treated with complete confidentiality and would not cause them any harm.

## Results

### Sociodemographic factors

A total of 570 participants took part in this study, with a response rate of 99.3%. The mean age of the women was 29 ± 6.65 years. Most of the women (94.9%) were married, and 299 (52.5%) were married between the ages of 18 and 24 years. Most respondents (98.4%) identified as Orthodox Christian. The majority of the women were housewives (69.5%). Of the women, 98 (17.2%) could not read and write, and 121 (21.2%) were in the poorest wealth index category (Table 2).

**Table 2:** Sociodemographic characteristics of women who gave birth in Shebel Berenta District, East Gojjam Zone, northeast Ethiopia, 2024 (*N* = 570).

Variables	Alternatives	Frequency	Percent
Age of marriage	≤17	175	30.7
	18–24	299	52.5
	≥25	96	16.8
Occupational status of husband	Farmer	357	67.0
	Merchant	66	12.4
	Daily laborer	23	4.3
	Government employee	87	16.3
Residence of respondent	Urban	51	8.9
	Rural	519	91.1
Women's occupational status	House wife	396	69.5
	Merchant	109	19.1
	Government employee	65	11.4
Educational status of the women	Cannot read and write	98	17.2
	Read and write informal	54	9.5
	Primary	272	47.7
	Secondary	83	14.6
	Higher or tertiary	63	11.1
Marital status	Married	541	94.9
	Divorced	29	5.1
Husbands’ educational status	Cannot read and write	76	14.3
	Read and write (informal	188	35.2
	Primary	161	30.2
	Secondary	35	6.6
	Tertiary or higher	73	13.7
Religion	Orthodox	561	98.4
	Muslim	9	1.6
Family have Community based health in insurance	Yes	408	71.6
	No	162	28.4
Family size	2–4	294	51.6
	5 and above	276	48.4
Time to reach health facility	<15 min	44	7.7
	15–30 min	96	16.8
	30 minutes to 1 h	187	32.8
	>1 h	243	42.6
Wealth index	Poorest	121	21.2
	Poor	92	16.1
	Medium	130	22.8
	Rich	113	19.8
	Richest	114	20.0

### Obstetric factors affecting ANC utilization among pregnant women

Of the women participating in this study, 436 (76.5%) were using modern family planning methods, of whom 78% used injectables. Of the pregnancies, 398 (69.8%) were planned. Among the participants, 216 (37.9%) were experiencing their first pregnancy. Of the mothers who had ANC contact, 403 (70.7%) started antenatal care late (after 12 weeks of gestation). More than half (53.2%) of the pregnant mothers had no awareness of danger signs during pregnancy. Of the mothers, 46 (8.1%) gave birth at home (Table 3).

**Table 3:** Obstetric characteristics of women who gave birth in Shebel Berenta District, East Gojjam Zone, northeast Ethiopia, 2024 (*N* = 570).

Obstetric characteristics		Frequency	Percent
Variables	Alternatives		
Number of pregnancies	First pregnancy	210	36.8
	2–4	229	40.2
	5 and above	131	23.0
Pregnancy time interval	<24 months	37	6.5
	24–34 months	191	33.5
	>34 months	120	21.1
Number of children alive	1–3	439	77.0
	≥4	131	23.0
Parity	Primipara	251	44.0
	Multiparity (2–4)	274	48.1
	Grand multipara (≥5)	45	7.9
Time of first contact initiation in week	Early initiated	167	29.3
	Late initiated	403	70.7
Number of ANC contact	1–3	156	27.4
	4–7	326	57.2
	8 and above	55	9.6
	No contact	33	5.8
Awareness on danger sign	Severe headache	28	8.9
	Blurring vision	111	35.5
	Vaginal bleeding	129	41.2
	Epigastria pain	31	9.9
	Decreased fetal movement	14	4.5
Awareness about danger sign	No	303	53.2
	Yes	267	46.8
Loss of pregnancy(abortion)	Yes	62	10.9
	No	508	89.1
Mode of delivery of last child	Spontaneous vaginal delivery	499	87.5
	Instrumental	59	10.4
	Cesarean delivery	12	2.1
Experience any complication during previous pregnancy, labor, and delivery	Yes	38	6.7
	No	532	93.3
Experience any complication during pregnancy for last child	Yes	86	15.1
	No	484	84.9
Pregnancy planned	Yes	398	69.8
	No	172	30.2
ANC contact for current child	Yes	537	94.2
	No	33	5.8
Place of delivery of last child	Home	46	8.1
	Health center	366	64.2
	Government hospital	158	27.7
Currently using FP	Yes	436	76.5
	No	134	23.5
Resumed mensuration after birth	Yes	390	68.4
	No	180	31.6
Type of family planning method used	Injectable	342	78.4
	Implants	94	21.6

### Knowledge and attitude toward ANC among pregnant women

More than half of the women (306, 53.7%) had poor knowledge about ANC contact, while the majority (94.4%) had a good attitude toward it.

### Health service and decision-making characteristics toward ANC among pregnant women

All women received antenatal care from government institutions during their pregnancy. Only 119 (20.9%) women made healthcare decisions independently, and 324 (56.8%) had no media exposure during their pregnancy (Table 4).

**Table 4:** Health service and decision-making characteristics of women who gave birth in Shebel Berenta District, East Gojjam Zone, northeast Ethiopia, 2024 (*N* = 570).

Variable	Alternatives	Frequency	Percent
By home decision made	By herself	119	20.9
	With husband	433	76.0
	With family	18	3.2
Having media exposure	Yes	246	43.2
	No	324	56.8
Place of ANC follow-up	Government institution	537	100

### Magnitude and distribution of eight or more ANC contacts among pregnant women

**Figure 1:**
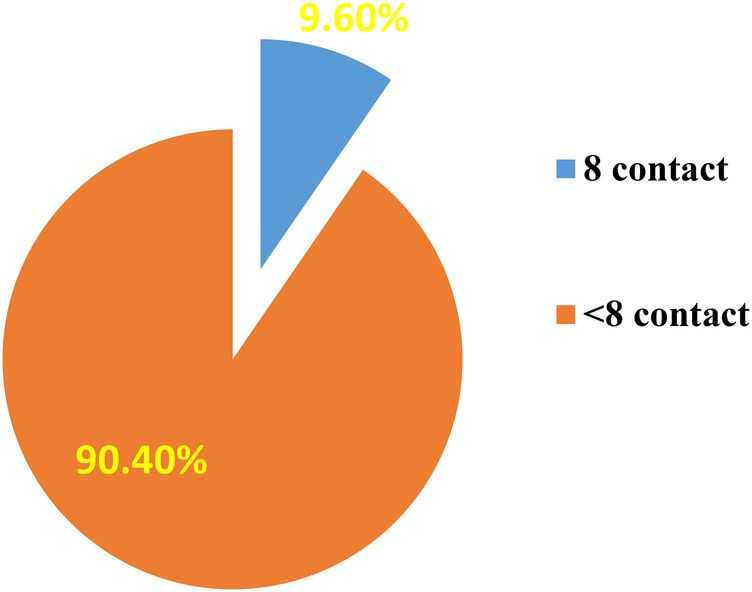
Magnitude of eight ANC contacts among women who gave birth in Shebel Berenta District, East Gojjam Zone, northeast Ethiopia, 2024 (*N* = 570).

**Figure 2:**
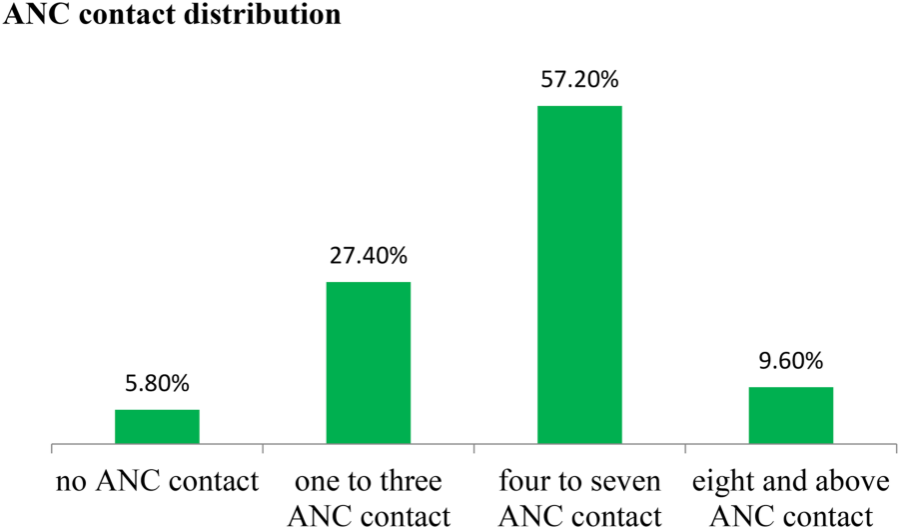
Distribution of ANC contacts among women who gave birth in Shebel Berenta District, East Gojjam Zone, northeast Ethiopia, 2024 (*N* = 570).

The prevalence of eight or more antenatal care contacts was 9.6% (95% CI: 7.4–12.3) among pregnant women (Figures 1, 2).

### Factors associated with eight or more ANC contacts among pregnant women

In the bivariate analysis, 12 independent variables were identified as candidates for multivariable analysis at a *p*-value of <0.25. These included knowledge about ANC, wealth index, number of pregnancies, family size, awareness of danger signs, timing of the first ANC contact, whether the pregnancy was planned, place of delivery, family planning use, media exposure, and women's occupation. All these variables were included in the multivariable analysis using the backward stepwise variable selection method. Knowledge about ANC, media exposure, timing of the first ANC contact, women's occupational status, number of pregnancies (gravidity), and family size were significantly associated with attending eight or more antenatal care contacts (*p*-value <0.05%; 95% confidence level).

In this study, women with good knowledge of ANC were 2.4 times more likely to have eight or more ANC visits, highlighting the importance of awareness in service utilization (AOR = 2.402; 95% CI: 1.115–5.175). Women who had media exposure were 2.47 times more likely to attend eight or more ANC visits compared to those who had no media exposure (AOR = 2.47; 95% CI: 1.15–5.306). Women who initiated the first ANC contact early were 5.64 times more likely to have eight or more ANC visits than those who started late (AOR = 5.46; 95% CI: 2.837–10.51). Women employed in government positions were 3.75 times more likely to have eight or more ANC visits than housewives (AOR = 3.745; 95% CI: 1.364–10.28). Women with two to four pregnancies were 3.524 times more likely to have eight or more ANC visits compared to primigravida women (AOR = 3.524; 95% CI: 1.696–7.32). In addition, women from households with fewer than five members were 3.005 times more likely to have eight or more ANC contacts than those with five or more family members (AOR = 3.005; 95% CI: 1.461–6.179). These factors were all significantly associated with completing eight or more ANC contacts (Table 5).

**Table 5:** Factors associated with eight ANC contacts among women who gave birth in Shebel Berenta District, East Gojjam Zone, northeast Ethiopia, 2024 (*N* = 570).

Variables	Category	Eight ANC contacts		COR (95% CI)	AOR (95% CI)	*p*-value
		Yes	No			
Knowledge	Poor knowledge	11 (20.0)	295 (57.3)	1	1	
	Good knowledge	44 (80.0)	220 (42.7)	5.36 (2.71–10.62)	2.40 (1.12–5.18)*	0.025
Number of pregnancies	First pregnancy	12 (21.8)	198 (38.4)	1	1	
	2–4	38 (69.1)	191 (37.1)	3.28 (1.67–6.47)	3.52 (1.70–7.32)**	<0.001
	≥5	5 (9.1)	126 (24.5)	0.66 (0.23–1.90)	1.89 (0.59–6.03)	0.281
Family size	2–4	41 (74.5)	253 (49.1)	3.03 (1.61–5.69)	3.01 (1.46–6.18)*	0.003
	5 and above	14 (25.5)	262 (50.9)	1	1	
Danger sign awareness	No	20 (36.4)	283 (55.0)	1	1	
	Yes	35 (63.6)	232 (45.0)	2.14 (1.20–3.79)	1.30 (0.63–2.68)	0.472
Time of ANC initiation	Early initiated	34 (61.8)	133 (25.8)	4.65 (2.61–8.29)	5.46 (2.84–10.50)**	<0.001
	Late initiated	21 (38.2)	382 (74.2)	1	1	
Planned pregnancy	Yes	47 (85.5)	351 (68.2)	2.75 (1.27–5.94)	1.44 (0.59–3.54)	0.425
	No	8 (14.5)	164 (31.8)	1	1	
Place of delivery	Home	3 (5.5)	43 (8.3)	1	1	
	Health center	27 (49.1)	339 (65.8)	1.14 (0.33–3.92)	0.89 (0.22–3.52)	0.867
	Hospital	25 (45.5)	133 (25.8)	2.69 (0.78–9.37)	1.72 (0.42–6.98)	0.449
Family planning use	Yes	47 (85.5)	389 (75.5)	1.90 (0.88–4.14)	2.01 (0.86–4.69)	0.108
	No	8 (14.5)	126 (24.5)	1	1	
Age of marriage	≤17	10 (18.1)	165 (32.0)	1	1	
	18–24	36 (65.5)	263 (51.1)	2.26 (1.09–4.67)	1.15 (0.49–2.70)	0.754
	≥25	9 (16.4)	87 (16.9)	1.71 (0.67–4.36)	2.34 (0.80–6.90)	0.122
Media exposure	Yes	41 (74.5)	205 (39.8)	4.43 (2.35–8.33)	2.47 (1.15–5.31)*	0.020
	No	14 (25.5)	310 (60.2)	1	1	
Women occupation	House wife	29 (52.7)	367 (71.3)	1	1	
	Merchant	18 (29.1)	91 (17.7)	2.500 (1.33–4.71)	1.864 (0.89–3.90)	.098
	Government employee	8 (18.2)	57 (11.1)	1.78 (0.77–4.08)	3.75 (1.36–10.28)*	.010
Wealth index	Poorest	10 (18.2)	111 (21.6)	1	1	
	Poor	13 (23.6)	79 (15.3)	1.83 (0.76–4.38)	1.76 (0.54–4.63)	0.406
	Medium	15 (27.3)	115 (22.3)	1.45 (0.62–3.36)	1.32 (0.48–3.61)	0.585
	Rich	12 (21.8)	101 (19.8)	1.32 (0.55–3.18)	0.71 (0.24–2.12)	0.540
	Richest	5 (9.1)	109 (21.2)	0.51 (0.17–1.54)	0.65 (0.15–2.81)	0.566

COR, crude odds ratio; AOR, adjusted odds ratio; CI, confidence interval; 1, reference category; *N*: frequency.

* *p* ≤ 0.05; **p < 0.001.

## Discussion

In this study, we investigated the prevalence and factors associated with achieving eight or more ANC contacts among women in Shebel Berenta District. The findings revealed that the prevalence of eight or more antenatal care contacts was 9.6% (95% CI: 7.4–12.3) among women who gave birth in the district. This result aligns with studies conducted in low- and middle-income countries using data from 54 DHS and Multiple Indicator Cluster Surveys (19), as well as studies from Benin and Cameron (25).

The prevalence in this study is higher than that reported in Bangladesh (26) and several African countries, including Mozambique, Mali, Guinea, Senegal, Uganda, and Zambia (25), as well as in Delta state in southern Nigeria (27), Rwanda (28), and Ethiopia (12). The difference might be related to variations in sociocultural factors, study settings, and periods.

The prevalence found in this study was lower than that reported in studies conducted in Nigeria (25, 29), Ghana (15, 30), and Liberia (31). The difference might be related to variations in sociocultural factors, study settings, and the timing of adoption and implementation of the newly recommended WHO antenatal care contact model. In Ethiopia, the new WHO model was only implemented at the end of 2022.

In this study, family size emerged as an important determinant of eight or more ANC contacts. Women from households with fewer than five members were three times more likely to complete eight or more ANC contacts compared to those from households with five or more members. This finding aligns with the DHS-based multi-level analysis conducted across 54 countries (19), as well as a study from Palestine (32). A possible explanation is that women in smaller households may have fewer domestic responsibilities and receive greater social and emotional support from their husbands and families, facilitating ANC visits.

Women with good knowledge about ANC were twice as likely to have eight or more ANC contacts compared to those with poor knowledge. This may be due to their greater understanding of the importance, timing, and frequency of ANC visits, which encourages them to seek appropriate healthcare and adhere to regular follow-ups during pregnancy. Good knowledge of ANC also allows women to recognize the risks associated with inadequate prenatal care, empowering them to prioritize their health and attend ANC appointments consistently.

Women exposed to media were twice as likely to have eight or more ANC contacts compared to those without media exposure, in line with findings from various studies (22, 27, 33, 34). This association may be explained by the role of mass media in promoting positive health-seeking behaviors through dissemination of information about the benefits of regular and timely ANC. Media also informs women about available services and health facility hours. Effective use of media platforms can improve maternal and child health outcomes by motivating women to seek and maintain ANC throughout pregnancy (26, 33, 34). Women with access to health information are generally more aware of the benefits of ongoing maternal healthcare, danger signs, and pregnancy-related complications than those without such access (35).

Women employed by the government were four times more likely to have eight or more contacts compared to housewives. This result is in line with evidence from 36 sub-Saharan African countries (33). This can be explained by government-employed women having better sociocultural attitudes toward the importance of ANC follow-up, stronger adherence to policy implementation, and generally more proactive health-seeking behaviors regarding ANC visits (33).

Women who initiated ANC contact early were five times more likely to have eight or more antenatal care contacts compared to those who started late. This result is consistent with studies conducted in Benin, Palestine, and Nigeria (32, 36). Early initiation allows for comprehensive monitoring of maternal health, timely detection of complications, and prompt interventions, which contribute to a higher number of ANC visits. In addition, women who start ANC early are more likely to receive essential health education on nutrition, lifestyle modification, birth preparedness, and danger signs during pregnancy. This fosters a stronger commitment to their pregnancy health and a positive attitude toward seeking healthcare, empowering them to actively participate in their pregnancy care and adhere to the recommended ANC visit schedule.

Women with two to four pregnancies were three times more likely to have eight antenatal care contacts compared to first-time pregnant women. This result contradicts with findings from multi-country representative data (25) and a study conducted across 36 sub-Saharan African countries (33). The discrepancy may be due to sociocultural differences, the time gap between studies, and the fact that women with multiple pregnancies tend to have greater experience and familiarity with the healthcare system and providers. Their increased knowledge and established perception of the importance of follow-up likely contribute to why women with two to four pregnancies are more likely to attend frequent ANC contacts compared to first-time mothers.

### Strengths and limitations of the study

The strength of this study lies in being the first in Ethiopia to use primary data after the implementation of the new antenatal care contact model aimed at improving maternal and child health outcomes. In addition, the community-based design allowed for the collection of adequate information.

However, as a community-based cross-sectional study, there is potential for recall bias, which may have led to an overestimation or underestimation of the prevalence of eight or more antenatal care contacts. Nevertheless, we attempted to ask questions related to the services received during antenatal care, including what was provided and how it was delivered.

## Conclusion

This study showed a low prevalence of eight or more antenatal care contacts. Key factors significantly associated with increased ANC visits included good knowledge about ANC, media exposure, early initiation of ANC, being a government employee, having two to four pregnancies, and a household size of fewer than five members.

These findings highlight the need for targeted interventions to improve antenatal care utilization. Strategies should focus on enhancing community education, expanding media campaigns on the importance of ANC follow-up, and raising awareness about relevant policies for the community.

## Data Availability

The original contributions presented in the study are included in the article/Supplementary Material, further inquiries can be directed to the corresponding author.
